# An Unusual Case of Gastrointestinal Bleeding in a Patient With COVID-19

**DOI:** 10.7759/cureus.13901

**Published:** 2021-03-15

**Authors:** Koushik Sanku, Abdul Hasan Siddiqui, Vishesh Paul, Moeez Ali

**Affiliations:** 1 Internal Medicine, Gandhi Medical College, Hyderabad, IND; 2 Pulmonary and Critical Care Medicine, University of Illinois at Urbana-Champaign, Champaign, USA; 3 Pulmonary and Critical Care Medicine, Carle Foundation Hospital, Urbana, USA; 4 Internal Medicine, California Institute of Behavioral Neurosciences & Psychology, Fairfield, USA

**Keywords:** covid 19, warfarin toxicity, corona virus, sars-cov-2, severe acute respiratory syndrome, lower gastrointestinal bleeding, bloody diarrhea, coagulopathy, anticoagulant therapy, case report

## Abstract

Coronavirus disease 2019 (COVID-19) is predominantly a respiratory disease that often presents with fever, cough, dyspnea, and myalgia or fatigue. Digestive symptoms such as nausea, vomiting, diarrhea, and abdominal pain may accompany respiratory symptoms. However, gastrointestinal (GI) bleeding among COVID-19 patients is a rare and unusual presentation, since these patients are frequently hypercoagulable and are less likely to bleed and more likely to clot. In this report, we present a case of an 80-year-old male with a history of type 2 diabetes mellitus, hypertension, and obesity who presented with GI bleed and was subsequently found to have COVID-19.

## Introduction

Coronavirus disease 2019 (COVID-19) patients usually present with typical clinical manifestations of fever, cough, dyspnea, and myalgia or fatigue [1]. The condition initially manifested as a respiratory disease and it still predominantly affects the respiratory system. However, over the last several months, many cases of COVID-19 affecting multiple organ systems have been reported, sometimes exclusively and sometimes together with respiratory involvement. After the respiratory system, COVID-19 has been most commonly associated with the gastrointestinal (GI) system. The most prevalent GI manifestations of COVID-19 include abdominal pain, nausea, vomiting, and diarrhea, with an incidence rate of 2-18.6%. But the incidence of digestive symptoms in critically ill patients has been reported to be as high as 36-50.5% [1,2]. However, GI bleed among COVID-19 patients has been less frequently reported in the literature, perhaps due to its frequently associated hypercoagulable pathophysiology. We present an unusual case of COVID-19 where the patient presented with GI bleed and an elevated international normalized ratio (INR). Interestingly, the anticoagulant state of this patient seemed to have played a protective role against the severe acute respiratory syndrome of COVID-19.

## Case presentation

An 80-year-old Caucasian male presented to the hospital with complaints of bloody stool and melena for a few days. The patient had a past medical history of pulmonary embolism in March 2015, status post-catheter-directed thrombolysis, and had been subsequently started on Coumadin for his unprovoked pulmonary embolism. He also had type 2 diabetes mellitus, obesity with a body mass index (BMI) of 31 kg/m^2^, hypertension, and chronic kidney disease (CKD) stage 1 with normal creatinine. During his current visit, the patient's blood hemoglobin level was found to have dropped to 7.4 g/dl from a last known value of 13.7 g/dl from around six months prior to the presentation, and his INR was elevated to more than 10 as he had overdosed on warfarin due to an accidental mix-up of doses in the past few days. The patient denied any significant abdominal pain, nausea or vomiting, or hematemesis. He denied any fever, chills, or night sweats, and also denied any cough or shortness of breath. The patient was admitted to the intensive care unit (ICU) for monitoring and transfusions. He received a unit of packed red blood cells, fresh frozen plasma, and IV vitamin K in addition to pantoprazole intravenously. A gastroenterology consult was placed for endoscopy and colonoscopy to identify the source of bleeding. However, due to COVID-19-related guidelines, a COVID-19 rapid antigen test was done, which was found to be positive. This was further confirmed using a reverse transcription-polymerase chain reaction (RT-PCR) test. Endoscopy and colonoscopy were deferred since the patient was hemodynamically stable, and he was closely monitored in the ICU for another day. Serial hemoglobin and hematocrit (H&H) monitoring and regular hemodynamic monitoring were done throughout his stay. The patient had no further bleeding episodes after the correction of INR, and his hemoglobin remained stable. The patient had no evidence of hypoxia or any respiratory symptoms, and a chest X-ray done in the ICU did not show any evidence of acute pulmonary disease. Therefore, he was eventually downgraded to the medical floor and subsequently discharged with instructions on safe warfarin dosing and monitoring.

## Discussion

GI manifestations are increasingly being reported among COVID-19 patients, with the most common symptoms being lack of appetite (78.6%), diarrhea (34%), vomiting (3.9%), and abdominal pain (1.9%) [1]. However, GI bleeding is still a rare manifestation and is usually seen in critically ill patients [1]. COVID-19 patients can present with GI bleeding as a result of direct or indirect mechanisms.

The primary cause of GI bleed is a direct injury to the GI mucosal cells from viral invasion [2]. Secondarily, there is increasing evidence that severe COVID-19 can be complicated with coagulopathy, which has a rather prothrombotic character and is associated with a high risk of venous thromboembolism (VTE) [3]. The endothelial cell dysfunction induced by infection results in excess thrombin generation and fibrinolysis shutdown, promoting a hypercoagulable state in patients with COVID‐19 [4]. Additionally, COVID‐19-induced hypoxia also increases blood viscosity and stimulates thrombosis via a hypoxia‐inducible transcription factor‐dependent signaling pathway [4]. These patients without thromboprophylaxis have an increased rate of mortality, and as many as 40% of such patients had eventually died [3].

Hence, therapeutic anticoagulation should be considered in patients hospitalized with COVID-19 [2]. In a recent study of 449 patients with severe COVID‐19, therapeutic anticoagulation with low molecular weight heparin (LMWH) appeared to be associated with a lower mortality rate in patients meeting sepsis‐induced coagulopathy (SIC) criteria, i.e., SIC scores of ≥4 (40.0% vs. 64.2%; p = .029) or those with D‐dimer levels of more than six-fold the upper limit of normal (32.8% vs. 52.4%; p = .017) [4,5]. However, despite the mortality benefit, these patients are likely to experience GI bleeding (estimated annual risk: 4.5-8%) as a complication of systemic anticoagulation [6].

Our case shed light on an unusual presentation of COVID-19. The patient had a GI bleed, which was evidently caused by warfarin toxicity. However, elevated INR seemed to have played a protective role in this patient from the respiratory manifestations of COVID-19. As per the existing evidence, elderly males, with hypertension, diabetes, and elevated BMI are at the highest risk of acquiring severe respiratory disease related to COVID-19. On the contrary, our patient had no definite manifestations of COVID-19. The patient had no significant disease from COVID-19 despite having all the risk factors, probably due to the benefits related to being on anticoagulants, which of course comes with the risk of increased bleeding incidence, especially in the elderly population. Although it can be argued that GI bleed in our case could be a manifestation of COVID-19 infection of the GI tract, we have no definitive evidence to substantiate this, and the finding of elevated INR in a patient on warfarin further supports the secondary cause of GI bleeding.

## Conclusions

GI bleeding can be one of the atypical presentations of COVID-19 infection. We discussed the case of a COVID-19 patient who presented with melena and supratherapeutic INR, which had possibly played a protective role against the severe respiratory disease that is commonly seen in elderly patients with COVID-19. This case report may indicate the potential benefit of anticoagulation in patients with COVID-19 in preventing severe respiratory disease in high-risk patients. Further research is needed to study how prophylactic/therapeutic anticoagulation may affect the severity of COVID-19 infection, as it can help better understand the pathophysiology of COVID-19 and formulate new treatment protocols for the future.
